# Similarity of drinking water biofilm microbiome despite diverse planktonic water community and quality

**DOI:** 10.3389/fmicb.2025.1567992

**Published:** 2025-06-18

**Authors:** Frances C. Slater, Katherine E. Fish, Joby B. Boxall

**Affiliations:** Sheffield Water Center, School of Mechanical, Aerospace and Civil Engineering, The University of Sheffield, Sheffield, United Kingdom

**Keywords:** drinking water distribution systems, microbiology, biofilms, community composition, metabarcoding

## Abstract

The impact of drinking water quality, in particular the planktonic microbiome, on the bacterial and fungal community composition of biofilms in drinking water infrastructure is explored. Understanding drinking water biofilms is critical as biofilms can degrade water quality and potentially present a public health risk if pathogens are released. Biofilms were developed for 12 months in three state-of-the-art pipe loop facilities installed at water treatment works and hence supplied by distinct treated drinking water and unique planktonic bacterial and fungal microbiomes. Each pipe loop had identical physical conditions, including pipe diameter, material and hydraulic regime (shear stress and turbulence). Despite the different bulk-waters, the bacterial and fungal community composition of the biofilm within each loop were remarkably similar, although in different quantities. The similarity between the biofilms from unique systems, with significantly different planktonic microbiomes, suggests shared selective pressures across the different sites which are independent of the varying water qualities, including planktonic community. This suggests that taking a global view of biofilm microbiome management is potentially feasible and that approaches controlling material or hydraulics may be best way to do this.

## 1 Introduction

Biofilms, consisting of microorganisms bound together in microbially derived extracellular polymeric substances (EPS), exist within drinking water distribution systems (DWDS), adhered to the pipe wall. As biofilms make up the vast majority of the microbial load within DWDS (Flemming et al., 2002) and impact water quality (Fish et al., 2017; Fish et al., 2020; Papciak et al., 2022), it is critical that biofilms are characterised and understood. Studies have characterised the planktonic microbiome (Perrin et al., 2019; Pinto et al., 2014; Zhang et al., 2017), and to a smaller extent the biofilm microbiome (Fish and Boxall, 2018), of drinking water systems.

A common assumption is that whilst planktonic and biofilm bacterial communities differ (Douterelo et al., 2013; Ling et al., 2016), the inoculum of the planktonic community has a dominant effect on the community composition of biofilms within DWDS (Kitajima et al., 2021; Liu et al., 2018). A large proportion of DWDS research focuses on planktonic analysis and omits the biofilm proportion of the DWDS microbiome, due to the ease and significant sampling complexity, respectively. Evidence suggests that DWDS biofilm growth does not plateau until biofilms are approximately 3 months old (Pick et al., 2021b). However, often studies are performed within bench top or laboratory scale systems, via short term experiments (≤1 month) and primarily analysing the planktonic community composition.

Many of these studies also only consider the bacterial community (Douterelo et al., 2013; Ling et al., 2016; Liu et al., 2018) despite DWDS communities being known to be complex and not only consist of bacteria but also fungi (Douterelo et al., 2018; Fish and Boxall, 2018), protozoa (Mai et al., 2023) and archaea (Kitajima et al., 2021). Fungi have previously been sampled to a lesser extent within operational DWDS, however more recently there is evidence of fungi being detected in drinking water internationally (Zhao et al., 2022), and evidence of fungi becoming incorporated into drinking water biofilms (Calero Preciado et al., 2021; Douterelo et al., 2018) and exhibiting increased tolerance to chlorine and mechanical stresses (Fish et al., 2020).

To understand any differences or similarities between planktonic and biofilm microbial communities, it is important to understand the environmental conditions within biofilms. Drinking water biofilms provide a selective advantage for microorganisms over their planktonic counterparts including increased nutrient availability (Denkhaus et al., 2006), and protection from disinfection (Ma et al., 2023) and hydraulic effects (Fish et al., 2022). Nutrients within DWDS follow a turbulence driven gradient to the pipe wall, resulting in a habitat that is less oligotrophic at the pipe wall (Morton et al., 2005).

Bench-top scale studies have found that organic and inorganic particles become incorporated within the EPS matrix of biofilms, providing a nutrient source to the biofilm community (Denkhaus et al., 2006). Due to the adsorption properties of EPS, natural organic matter found in water can also accumulate in biofilms (Lemus-Perez and Rodriguez Susa, 2020). Whilst such laboratory based experimental set-ups go some way to understanding the impact of nutrients on DWDS biofilms, bench-top scale reactors do not accurately reflect the spatial and temporal changes in nutrients, particularly AOC concentration, or the hydraulic conditions within the DWDS environment. DWDS are highly complex systems comprising both temporal variations including temperature, and spatial variations such as different pipe materials, dimensions and surface properties. Pick et al. (2021a) showed the impact of assimilable organic carbon (AOC) concentration within the bulk-water on biofilm growth and mobilisation using state-of-the-art pipe loop facilities installed within operational water treatment works (WTW). AOC is the fraction of carbon most easily assimilated by heterotrophic microorganisms for growth, resulting in an increase in cell numbers. To date, the impact of the AOC concentration on the microbiome of drinking water biofilms in systems representative of full-scale DWDS infrastructure, including water supply and pipe materials, has not been explored.

Conversely, disinfection can react with organic nutrients, with the disinfectant concentration in the bulk-water reducing in concentration at the pipe wall (Ma et al., 2023).

The EPS matrix has been reported to provide microorganisms with protection from multiple environmental stresses in the water column including disinfection (Wang et al., 2014). Fish et al. (2020) used a full-scale DWDS experimental facility to determine the impacts of different free chlorine regimes on biofilm EPS composition, structure and microbiome. Bacterial communities within the biofilms were found to be distinct between the three chlorine regimes, with biofilms exposed to high chlorine concentration being found to contain bacteria tolerant to oxidants and therefore less likely to be inactivated by the chlorine residuals (Fish et al., 2020). In contrast, the fungal biofilm communities were found to be unique but did not follow a chlorine dose response, therefore exhibiting the importance of analysing both taxa. Comparisons of planktonic and biofilm microbiome were not possible as the planktonic community was not assessed in this study (Fish et al., 2020).

Hydraulic conditions vary both temporally and spatially, and have been demonstrated to impact biofilms (Fish et al., 2017; Fish et al., 2022). Temperature is a key parameter influencing drinking water microbial community composition (Calero Preciado et al., 2021; Pinto et al., 2014). Pinto et al. (2014) conducted a 15-month survey of planktonic bacterial community dynamics within a DWDS and found that the patterns in spatial dynamics were weaker than those for the temporal trends, with community composition being correlated with changes in temperature and source water.

This research aimed to understand to what extent the planktonic microbiome seeds drinking water biofilms, or alternatively determine if it is environmental factors within the DWDS itself that govern the biofilm microbiome. Understanding this is critical to ascertaining how transferable management approaches are between systems with different planktonic communities. This research therefore aimed to understand how differences in bulk-water qualities, influenced the planktonic and biofilm microbiome (and interactions between the two) within DWDS facilities. Biofilms were grown in three DWDS facilities supplied by treated drinking water for 1 year, with in-depth planktonic and biofilm microbiome being conducted to determine any changes in community composition.

## 2 Methods and methodology

### 2.1 Experimental overview

**Figure 1:**
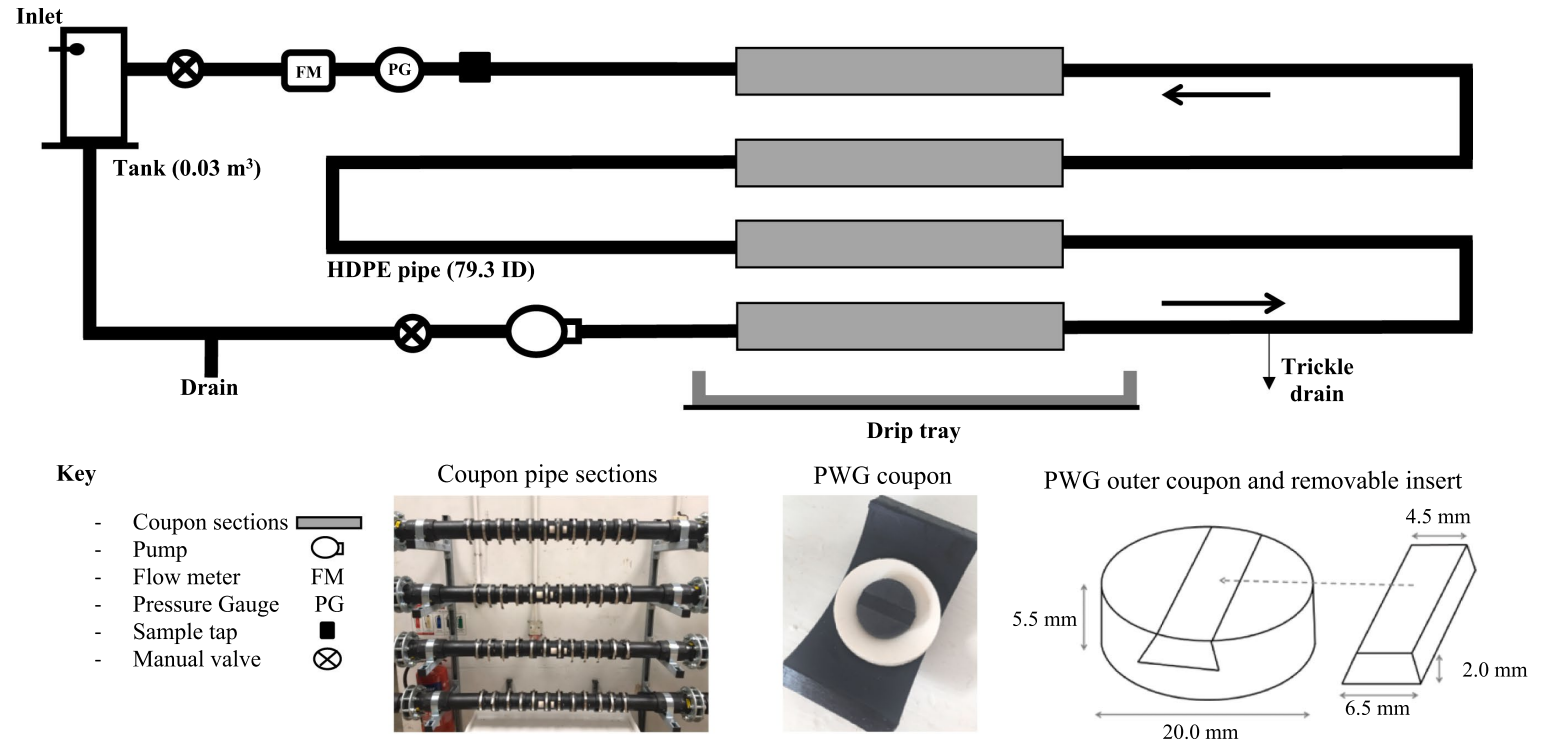
Schematic of pipe loop experimental facility (Pick et al., 2021a, 2021b), with photographs showing coupon sections and the Pennine Water Group coupons used for biofilm sampling. Entire loop 10 m in length consisting of high-density polyethylene (HDPE) PE100 pipe with a 79.3 mm internal diameter. The inlet is a pressurised supply, and greater than the loop pressure to prevent back flow.

In order to explore the impact of bulk-water quality on biofilm community (bacteria and fungal) compositions under conditions representative of full-scale DWDS infrastructure, experiments were undertaken within three experimental pipe loop facilities at operational WTW, as described in Pick et al. (2021a) and shown in Figure 1. Pipe loop facilities act as a scaled down version of a drinking water network, providing a suitable environment for representative biofilm sampling whilst mimicking the hydraulic and water quality conditions found within an operational DWDS. The facilities were operated for a one-year longitudinal study to include seasonal impact on water quality. Each pipe loop replicated the system retention time (24 h), water chemistry and microbiology of operational DWDS, whilst enabling laboratory level control and biofilm sampling. This overcomes the limitations of bench-top scale studies, but minimising the risks, lack of control and near impossibility of obtaining representative, uncontaminated biofilms samples from operational systems. In order to be able to isolate the impact of the different bulk-water quality parameters on biofilm community composition, the hydraulic conditions, pipe material and temperature were the same across the experimental facilities. Biofilms were grown simultaneously for 1 year at each site (May–May), then exposed to elevated shear stress (mimicking a hydraulic event) to evaluate the biofilm response and impact on water quality.

### 2.2 Site selection and pipe loops

Three distinct WTW sites were selected to enable comparisons of different water qualities on the biofilm microbiome, as shown in Table 1. All WTW sites are encompassed within the a 36 mile radius of each other, but are within distinct watersheds.

**Table 1:** Site details for the water supplying the three full-scale experimental pipe loops.

Site	Source water	Treatment type	Residual disinfectant type
A	Surface (reservoir)	RGF	Cl_2_
B	Surface (river)	Membrane	NH_2_Cl
C	Groundwater		Cl_2_

RGF, rapid gravity filter; Cl_2_, chlorine; NH_2_Cl, monochloramine.

The DWDS experimental pipe loops installed at each of the three sites were built to identical design specifications, as shown in Figure 1. Each pipe loop consisted of a 10 m long length of high-density polyethylene (HDPE) PE100 pipe with a 79.3 mm internal diameter. Pennine Water Group (PWG) coupons (Deines et al., 2010) with a curved outer coupon section and flat insert piece were installed in each DWDS pipe loop to provide a removable surface for biofilm sampling that caused minimal distortion of the hydraulic boundary layer. Each loop had four straight coupon sections, each section containing 12 coupons (48 in total). Any minimal drips leaking from the coupons were captured by the drip tray and combined and measured with the trickle drain flow to accurately set the system residence time. Online turbidity meters (ATI A15/76, Analytical Technology Inc., United Kingdom—see Supplementary Table 1) were connected to the sample tap during the mobilisation ‘flushing’ phase. HDPE pipe was selected as it is representative of the pipe material most commonly installed in the UK. Although less common by length than cast iron, HDPE it is increasingly used for new pipe installations and it has far more consistent material properties than cast iron. There is an observed increase in bursts and subsequent repair rates during and post-world war cast iron pipes when material was scarce and low quality. Cast iron can also degrade and corrode over time (John et al., 2024), which is very different as a function of the water type.

### 2.3 Biofilm growth

Biofilms were grown in the pipe loops for 1 year under a constant flow rate of 0.4 L/s (shear stress 0.03 Nm^−2^, Re 4,947). This flow rate was selected as the average flow rate in 75–100 mm diameter pipes within UK DWDS, as stated by Husband et al. (2008).

Treated drinking water specific to each site was re-circulated around each experimental system from an enclosed, 30 L (0.03 m^3^) tank, via a variable speed pump. A system residence time of 24 h was set using a trickle-feed and drain to provide representative water quality of each DWDS, and preserve a baseline nutrient supply and disinfection residual, among other water quality parameters. Note this system residence time is independent of the recirculation time. Each parcel of water circulated through the pipe and back to the tank almost 700 of times over its 24-h residence time (recirculation time of 125 s).

The loops were newly purpose built for the experiment. To further ensure consistent initial conditions, each pipe loop was disinfected immediately prior to the experiment. This comprised of 24 h hyper chlorination with a 20 mg L^−1^ concentration of a sodium hypochlorite solution (VWR International Ltd., United Kingdom) (11–14% free chlorine), which was re-circulated within the system at a maximum flow rate of 5.0 L/s. After 24 h, each pipe loop was repeatedly flushed (to waste) repeatedly at the maximum flow rate with treated drinking water specific to each site, until chlorine levels decreased to those of the inlet water, prior to the biofilm growth phase starting.

### 2.4 Mobilisation of biofilm

Following the 12-month growth period, the pipe loops were ‘flushed’ (closed loop, no trickle drain or feed) and the response observed (biofilm and bulk-water). A series of flushing steps (1.5, 2.5, 3.5 and 5.0 L/s with boundary shear stress steps of 0.33, 0.83, 1.55 and 3.02 Nm^−2^) were used to gain deeper insight into the mobilisation of the developed biofilm material as a function of imposed shear stress. Coupon sampling after this sequential flushing enabled examination of the biofilm microbiome remaining to regrow.

### 2.5 Biofilm and planktonic sampling

Biofilm samples were collected in triplicate at day 0 (*n* = 3), 3 months (*n* = 3), 6 months (*n* = 3), 9 months (*n* = 3), 12 months (*n* = 3) and once following the mobilisation (post-flush) (*n* = 3). Day 0 samples were defined as coupons which were in the pipe loop for ≤90 min after the system clean. To facilitate biofilm sampling and coupon removal the pump was briefly stopped, and the relevant manual valves closed. These stoppages were of short duration and valve and pump operations were slow to avoid transients and minimise impacts. Coupons were positioned along either side of the pipe length, with the exception of nine top or bottom positioned coupons. The top and three bottom positioned coupons were used to assess any difference in biofilm accumulated at either position, after 12 months growth and following the mobilisation phase (post-flush). Coupons were carefully removed from each pipe and the outer coupon was separated from the insert using sterilised forceps. The coupon inserts were used in a parallel study (Pick et al., 2021a).

The biofilm was removed from the coupon and homogenised by placing the coupon in a petri dish with 30 mL of sterile phosphate buffer and repeatedly brushed using a sterile toothbrush (Pick and Fish, 2024). A 1 mL volume of each biofilm suspension was used for total and intact cell count (TCC and ICC) analysis, and the remaining sample was filtered for microbial community composition analysis.

In order to compare the planktonic and biofilm communities, water samples (*n* = 3) were collected from each pipe loop. Water samples (1 L) were taken directly from the sample tap of each loop at day 0, 6 months and 12 months. A total of 54 biofilm suspensions and 27 water samples were collected from the three facilities and filtered through 0.22 μm pore nitrocellulose filter (Millipore, MA, United States) using a Microstat membrane filtration unit (Sartorius, United Kindom). Filters were then stored in sterile bags at −80°C prior to DNA extraction.

### 2.6 Water quality analysis

Throughout the 12-month growth phase, bulk-water quality was measured in triplicate every 2 weeks capturing: AOC, TOC, total & free chlorine, turbidity, iron, manganese, temperature, and pH. This was done within each pipe loop and the post-treated water supply from each WTW feeding each pipe loop. A summary of the instruments and methods used for measuring discrete bulk-water parameters during both the growth and the mobilisation experimental phases is provided in Supplementary Table 1. AOC measurements were conducted using the AOC method developed in Pick et al. (2019).

### 2.7 Biofilm and planktonic microbiome analysis

#### 2.7.1 Cell quantification

Planktonic and biofilm TCC and ICC (cells/mL or cells/mm^2^) were measured using the flow cytometry method detailed in Gillespie et al. (2014). In summary, water samples were dechlorinated with sodium thiosulphate before 500 μL water samples were stained with 5 μL SYBR Green (Life Sciences, California, United States) for TCC. For ICC, an additional 500 μL volume of the water sample was stained with 6 μL SYBR Green/Propidium Iodine mixture (Life Sciences, California, United States), with a final concentration of 1× SYBR Green and 3 μM PI. Biofilm TCC and ICC were obtained using the same staining protocol and converted to cells/mm^2^. All samples were analysed using BD Accuri C6 Flow Cytometer (BD Accuri, United Kingdom), with fixed gates as per Fish et al. (2020). All appropriate negative controls were performed, including negative controls for stains, and calibration beads were run daily.

#### 2.7.2 DNA extraction and Illumina sequencing

DNA was extracted from the nitrocellulose filters using DNeasy PowerSoil Kit (Qiagen, Germany). In addition to the samples, “biofilm control” filters were also exposed to the DNA extraction process and “DNA controls” were run: empty sterile tubes to which all the solutions were added and all the processes applied. All biofilm control filters were found to be negative. Only two of the three day 0 site B, and two of the three day 0 site C biofilm samples contained quantities of bacterial or fungal DNA detectable via the methods used in this study. DNA was successfully extracted from three day 0 biofilm samples from site A. DNA was successfully extracted from all other samples.

PCR cycles were conducted using the HotStarTaq Plus Master Mix Kit (Qiagen, United States). The following protocol was carried out which included: 94°C for 3 min, followed by 30 cycles of 94°C for 30 s, 53°C for 40 s, 72°C for 1 min, and a final elongation step at 72°C for 5 min. APCR products were checked using a 2% agarose gel.

Sequencing was carried out on a Illumina MiSeq platform using the paired-end protocol by Mr. DNA Laboratory (TX, United States). The bacterial 16 s region was amplified using primers 28F (5′-GAGTTTGATCNTGGCTCAG-3′) and 519 (5′-RGTNTTACNGCGGCKGCTG-3′) spanning the V1 to V3 hypervariable regions., and the fungal 18S rRNA gene was amplified using primers SSUFungiF (5′-TGGAGGGCAAGTCTGGTG-3′) and SSUFungiR (5′-TCGGCATAGTTTATGGTTAAG-3′) targeting the ITS1-2 regions (Hume et al., 2012). Raw MiSeq data have been uploaded to the NCBI Sequence Read Archive under accession number PRJNA1273128.

### 2.8 Data analysis: water quality

The mean, median, range and standard deviation were calculated for each of the parameters listed in Supplementary Table 1 during the growth phase. The normality of the data was analysed using the Shapiro–Wilks test and parametric (ANOVA and Tukey) or non-parametric tests (Kruskal Wallis and two-sample Wilcoxon), as appropriate, to identify any differences in water quality parameters between experiments. Data collected during the mobilisation phase was plotted against shear stress and a linear model and regression analysis was performed to identify relative changes (each loop was analysed separately). The *R*^2^ and *p*-values were used to assess the fit of the linear model to the data and the significance of the gradient to determine which parameters responded significantly to the elevation in shear stress. All statistical analysis and graphical plots were generated in R v4.4.1 (R Foundation for Statistical Computing Platform, 2018) with a significance level of <0.05.

### 2.9 Data analysis: bioinformatics

Sequences were depleted of barcodes and primers, then sequences <150 bp, with ambiguous base calls and with homopolymer runs exceeding 6 bp were removed from further analysis. Sequences were denoised and operational taxonomic units (OTUs) generated. The data underwent quality filtering, trimming, removal of chimeras, and truncation of low-quality reads using the DADA2 plugin. OTUs were defined by clustering at 3% divergence (i.e., 97% similarity cut off) and selected using UPARSE (Edgar, 2013). After quality filtering and taxa classification, a total of 2,245,863 16S rRNA gene sequences were obtained from 81 samples. Taxonomic assignments were made with USEARCH global alignment program (Edgar, 2013). OTUs were taxonomically classified using BLASTn against a database derived from NCBI.^1^ Biofilm samples for taxonomic analysis are labelled in which the first letter/number indicates time point, the second letter indicates the site (A, B or C), and the third number indicates the triplicate number.

Bacteria and fungi presence/absence and relative abundance data were analysed using PRIMER-6 (v6.1.13, PRIMER-E Ltd., United Kingdom) for multi-variate analysis. Data was transformed (square root) and Bray–Curtis analysis was performed to generate similarity matrices (visualised via non-metric multi-dimensional scaling—nMDS) and enable hierarchical clustering analysis (visualised via dendograms). All nMDS plots were generated using 400 iterations of the data and the stress values for 2D plots noted (stress <0.05 = excellent representation of data, <0.1 = good representation, >0.3 = weak representation). Cluster analysis was run for 20,000 permutations and a dendrogram plotted. In dendogram plots, sample identification numbers are shown (first letter/number indicates time point, the second letter indicates the site, and the third number indicates the triplicate number). Red lines in dendogram plots indicate profiles not significantly dissimilar according to SIMPROF analysis.

Resemblance of communities between sites, time points and sample type (planktonic or biofilm) were assessed for statistically significant differences using analysis of similarities (ANOSIM). ANOSIM analyses (one-way and two-way) were run with a maximum of 400,000 permutations, the global-*R* values (0 = same, 1 = completely different) and *p*-values (<0.05 significant; >0.05 weak evidence) are reported. The global-*R* statistic values represent the strength of the impact that the factors analysed had on the samples. Similarity percentage analysis (SIMPER) was used to evaluate the similarity between replicates and sample groups (expressed as %), and conducted on the different taxonomic-level datasets to ascertain the genera mainly responsible (threshold of ≥75%) for driving differences between the sites, time points or sample types.

Ecological indices were used to assess community structure, specifically the relative richness, the relative diversity, determined using the Shannon index (Shannon, 1948), and relative evenness, generated using the Pielou index (Pielou, 1966). The relative ecological indices were exported from PRIMER 6 and analysed using R v3.5.2 (R Foundation for Statistical Computing Platform, 2018) to determine statistically significant similarities/differences [via *t*-tests or analysis of variance (ANOVA)].

## 3 Results

### 3.1 Bulk-water quality

To assess the bulk-water quality supplying the three pipe loop facilities, UK regulated drinking water parameters were monitored along with AOC, TCC, and ICC (Table 2). Average planktonic TCC and ICC were highest within site A (surface reservoir), then site B (surface river) and then C (ground-water) lowest, and statistically different (TCC: *χ*^2^ = 584.08, DF = 2, *p* = <0.001; ICC: *χ*^2^ = 446.64, DF = 2, *p* = <0.001). Chlorine residual type differed between sites (site A and C free chlorine; site B chloramine) and residual chlorine concentrations were statistically different between the sites (free chlorine: *χ*^2^ = 53.976, DF = 2, *p* = <0.001). Turbidity, iron, manganese, TON and phosphate were not statistically different (*p* > 0.05) between all three sites. The AOC concentration was highest within site A, mid-range at site B and lowest at site C and significantly different (AOC: *χ*^2^ = 416.82, DF = 2, *p* = <0.001). TOC was lower at site C than A or B. The AOC concentration within site C showed least seasonal variation, consistent with behaviour of ground source waters. The range of seasonal temperature at site A and B were similar as they are both surface water sources. Site C, a ground-water source, had a slightly lower mean temperature (10.9°C) and exhibited less standard deviation, despite the 24 h residence time in the pipe loop.

**Table 2:** Bulk-water quality within each pipe loop during the formation of biofilms over the 12 month period at site A, site B, and site C.

Water quality parameter	Pipe loop A mean (SD^a^)	Pipe loop B mean (SD^a^)	Pipe loop C mean (SD^a^)
TCC (cells/mL)*	93,017 (83,662)	359 (170)	243 (98)
ICC (cells/mL)*	354 (190)	70 (46)	63 (140)
Total chlorine (mg/L)*	0.90 (0.09)	1.34 (0.09)	0.62 (0.03)
Free chlorine (mg/L)*	0.87 (0.10)	0.12 (0.020)	0.50 (0.04)
Turbidity (NTU)	0.01 (0)	0.05 (0.02)	0.01 (0)
AOC (μg C/L)*	290 (31)	279 (35)	71 (12)
Water temperature (°C)*	11.07 (5.3)	11.7 (4.8)	10.9 (4.1)

Replication *n* = 3, samples collected fortnightly for 12 months = 72 samples. SD, standard deviation; AOC, assimilable organic carbon; TCC, total cell count; ICC, intact cell count; NTU, nephelometric turbidity units. Parameters marked with * were statically different between sites.

Comparison of the values in Table 2; Supplementary Table 2 reveals that the water quality in each of the pipe loops was representative of post-treated (final) water entering each of the DWDS (not significantly different), and therefore any samples obtained from the pipe loops were representative of the network biofilm and planktonic microbiome. The greatest difference in values is seen in the cell count data, increasing due to the 24 h residence time in the loops at sites A and B. This correlates with a slight decrease in AOC at these sites. Interestingly there is no measurable decrease in disinfection residual associated with this regrowth and the significant residual values did not inhibit this regrowth.

### 3.2 Planktonic microbiome

#### 3.2.1 Diversity indices of bacterial and fungal planktonic communities

Diversity indices including relative richness, relative evenness and relative diversity were calculated for bacterial (Table 3) and fungal communities (Table 4) to understand and quantify the differences in the community structure of the water circulating around each pipe loop. The relative richness of bacteria within bulk water at day 0, month 6, and month 12 were found to exhibit site-specific trends, with bacterial richness increasing over time at site A, but decreasing from day 0 to month 6 at sites B and C. Site location had a statistically significant effect on the relative richness of bacterial planktonic communities at day 0 (*p* < 0.001), month 6 (*p* = 0.02), and month 12 (*p* = 0.04). The relative evenness of planktonic bacterial communities was found to exhibit little change over time or between sites and was not statistically different. The relative diversity of bacterial communities exhibited the inverse trend to relative richness, with a declining trend in diversity from month 6 to month 12 at site A, and an increase in diversity at site B over time.

**Table 3:** Ecological indices of bacterial planktonic communities from drinking water bulk water samples supplying pipe loops A, B & C (*n* = 3) at day 0, month 6, and month 12.

Site	Time	Relative richness (Chao 1)		Relative evenness (Simpson)		Relative diversity (Shannons index)	
		Mean	StDev	Mean	StDev	Mean	StDev
A	Day 0	1362.00	85.98	0.89	0.01	5.82	0.52
	Month 6	1251.33	184.32	0.88	0.04	5.85	0.71
	Month 12	1569.67	97.90	0.86	0.04	5.26	1.00
B	Day 0	911.00	30.27	0.94	0.01	3.89	0.55
	Month 6	796.00	61.51	0.93	0.01	4.27	0.28
	Month 12	914.67	57.83	0.94	0.01	4.51	0.13
C	Day 0	622.67	127.01	0.98	0.02	3.20	0.17
	Month 6	566.00	110.57	0.98	0.01	3.73	0.44
	Month 12	710.33	83.63	0.99	0.01	3.63	0.45

(A) Relative richness, (B) relative evenness, and (C) relative diversity.

**Table 4:** Ecological indices of fungal planktonic communities from drinking water bulk water samples supplying pipe loops A, B & C (*n* = 3) at day 0, month 6, and month 12.

Site	Time	Relative richness (Chao 1)		Relative evenness (Simpson)		Relative diversity (Shannons index)	
		Mean	StDev	Mean	StDev	Mean	StDev
A	Day 0	1005.67	15.82	0.89	0.03	4.84	0.68
	Month 6	1013.67	31.63	0.86	0.01	5.21	0.35
	Month 12	994.67	54.15	0.90	0.02	4.69	0.71
B	Day 0	755.67	116.32	0.94	0.01	3.87	0.41
	Month 6	871.67	53.78	0.95	0.02	4.15	0.28
	Month 12	783.33	49.00	0.94	0.01	3.96	0.42
C	Day 0	651.67	72.25	0.97	0.00	3.30	0.28
	Month 6	686.67	75.08	0.99	0.01	3.51	0.29
	Month 12	646.33	61.08	0.99	0.01	3.13	0.10

(A) Relative richness, (B) relative evenness, and (C) relative diversity.

The relative richness and diversity of fungal planktonic communities (Table 4) was found to increase from day 0 to month 6, before declining at month 12 at all sites. Similar to the planktonic bacterial communities, the relative evenness of planktonic fungal communities was found to remain consistent over time and between sites.

#### 3.2.2 Bacterial planktonic community composition

**Figure 2:**
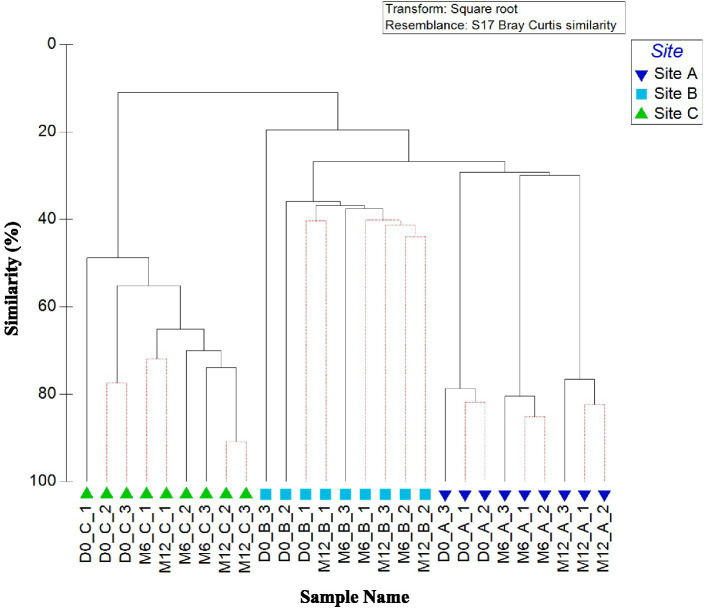
Variation between planktonic bacterial communities at the three sites over 1 year. Dendrogram based on Bray–Curtis similarities using OTU relative abundance data. Sample names are written in the format of time point_site_replicate. Time points are day 0 (D0), month 6 (M6) or month 12 (M12). Numbers 1, 2, and 3 (A–C) highlight distinct clusters. Red lines indicate profiles not significantly dissimilar according to SIMPROF analysis.

Figure 2 shows the similarities in bacterial community between all water samples (day 0, month 6, and month 12), analysed at the OTU level. A clear difference is apparent between the planktonic bacterial community composition in the three pipe loops (as indicated by clusters 1, 2 and 3 on Figure 2). Water within the pipe loop at site A was the only site to exhibit a significant change in bacterial community over all time points (Clusters 3a, 3b, and 3c, Figure 2).

ANOSIM values confirm that the differences in bacterial community between sites was statistically significant for all relative abundance data (*p* < 0.05). ANOSIM data by site showed that presence/absence data had, on average, a lower global *R* value (*R* = 0.532) than relative abundance data (*R* = 0.991). SIMPER analysis identified that average similarity of 12-month relative abundance data was highest between site A and B (average similarity of 16.14) and lowest between site A and C (average similarity of 9.26).

**Figure 3:**
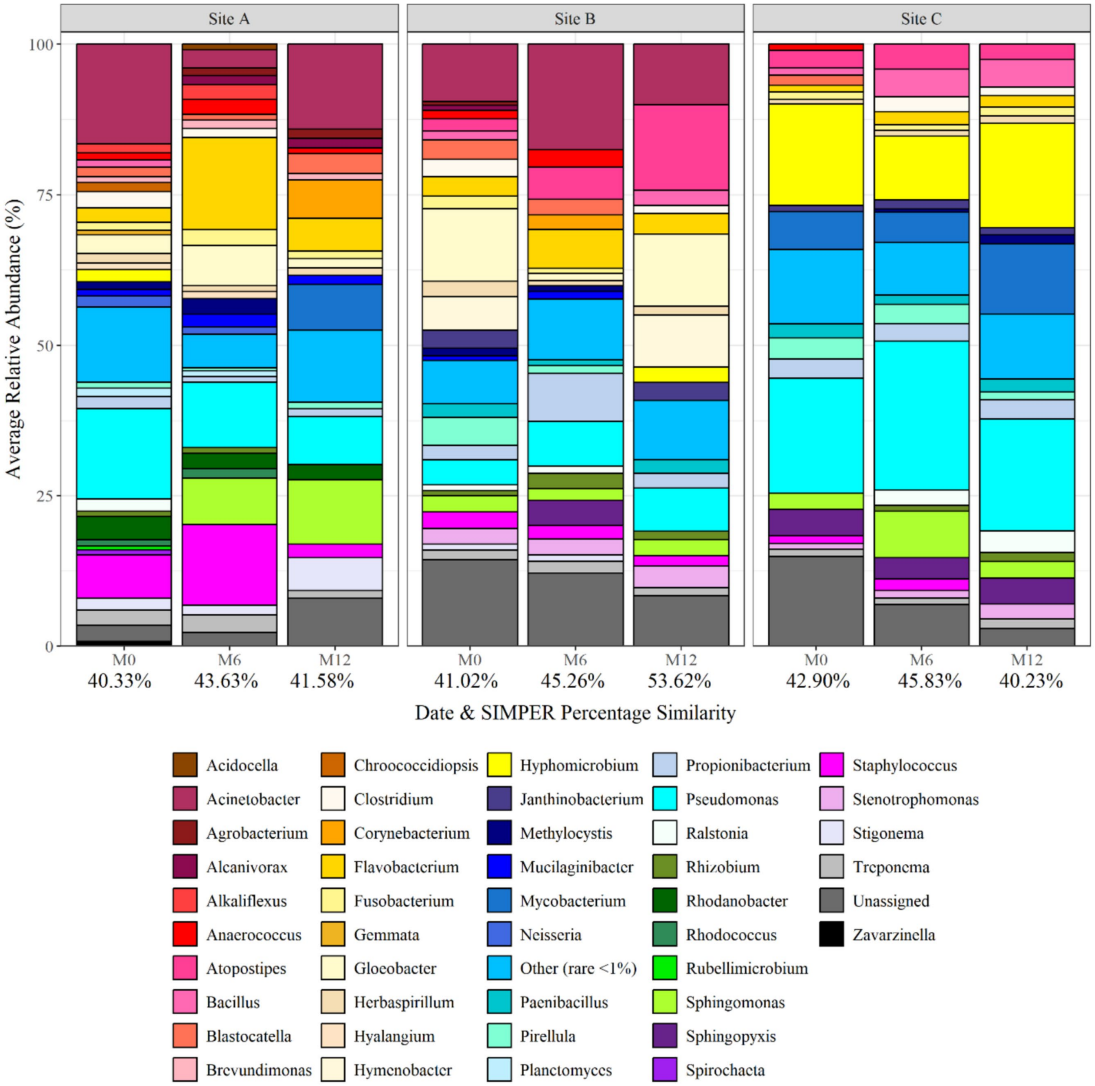
Bacterial planktonic community composition at each site and over time at genus level. Numbers below each sampling day (day 0, month 6, and month 12) indicate the average replicate similarity (each bar is the average of three repeat samples). Data responsible for >1% relative abundance plotted.

Figure 3 shows the average relative abundance of the different planktonic communities at genus level, showing spatial and temporal variation. The dominant bacterial class was *Proteobacteria* at all sites and times. The dominant genera included *Pseudomonas*, *Hyphomicrobium*, *Acinetobacter*, *Staphylococcus*, *Hymenobacter* and *Gloeobacter*, with the specific dominant genus varying with time and site. *Pseudomonas* was particularly dominant at site C, being responsible for 19% of the average relative abundance at month 12. *Acinobacter* was found to be present at site A and B (both supplied by surface water) throughout the 1 year study, but absent from site C (ground-water site). In addition, *Hyphomicrobium* was found in greatest abundances within site C, being responsible for 17, 10, and 17% of the average relative abundance at site C at month 0, 6, and 12, respectively. SIMPER analysis was applied to month 0, month 6, and month 12 planktonic samples to identify the bacterial taxa at genus level driving the differences between the communities between sites. Of the genera detected 10, 27, and 22 explained 75% of the difference between the sites at month 0, month 6, and month 12, respectively. Among the differentiating genera were *Acinobacter*, *Hymenobacter*, and *Gloeobacter*. *Hymenobacter* was found to only be present at site B which was the only chloraminated site.

#### 3.2.3 Fungal planktonic community composition

**Figure 4:**
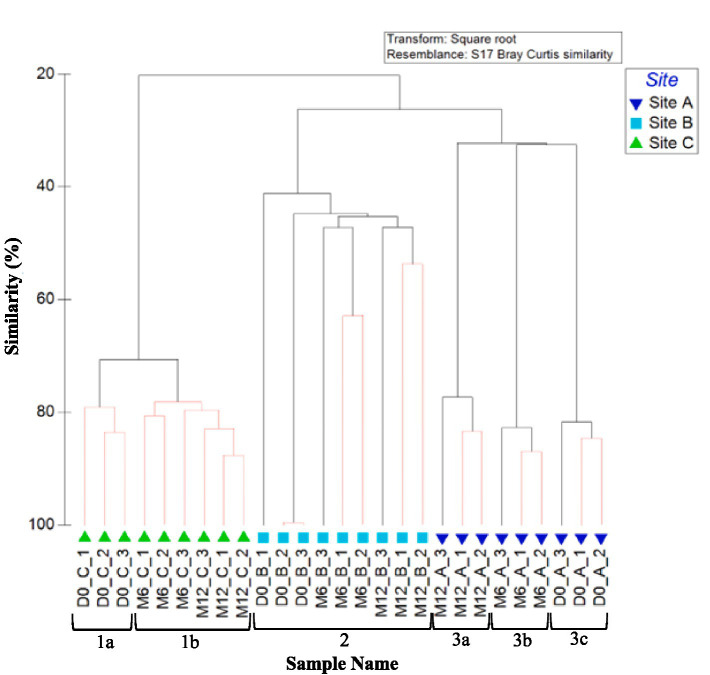
Variation between planktonic fungal communities at the three sites over 1 year. Dendrogram based on Bray–Curtis similarities using OTU relative abundance data. Sample names are written in the format of time point_site_replicate. Time points are day 0 (D0), month 6 (M6) or month 12 (M12). Numbers 1, 2, and 3 highlight distinct clusters. Red lines indicate profiles not significantly dissimilar according to SIMPROF analysis.

Figure 4 shows the similarities in planktonic fungal community at each site (day 0, month 6, and month 12), analysed at the OTU level. As found with planktonic bacterial OTU relative abundance data (Figure 2), samples group by site. Also as was seen for the bacterial communities, site A exhibited a change in fungal community composition with time, with three distinct clusters at day 0, month 6, and month 12 (Figure 4; clusters 3a, b, and c). A distinct cluster was also identified at day 0 within site C (Figure 4; cluster 1a), but month 6 and month 12 data were unable to be distinguished from each other.

ANOSIM values confirm that the site difference in fungal relative abundance was statistically significant (*R* = 0.651, *p* = 0.001). When comparing day 0, month 6, and month 12 relative abundance data, site A had the highest ANOSIM global *R* value (*R* = 0.524, *p* = 0.020), suggesting site A planktonic communities differed the most with time. There were no significant differences in fungal community with time at site B and site C (*R* = 0.235, *p* = 0.126).

**Figure 5:**
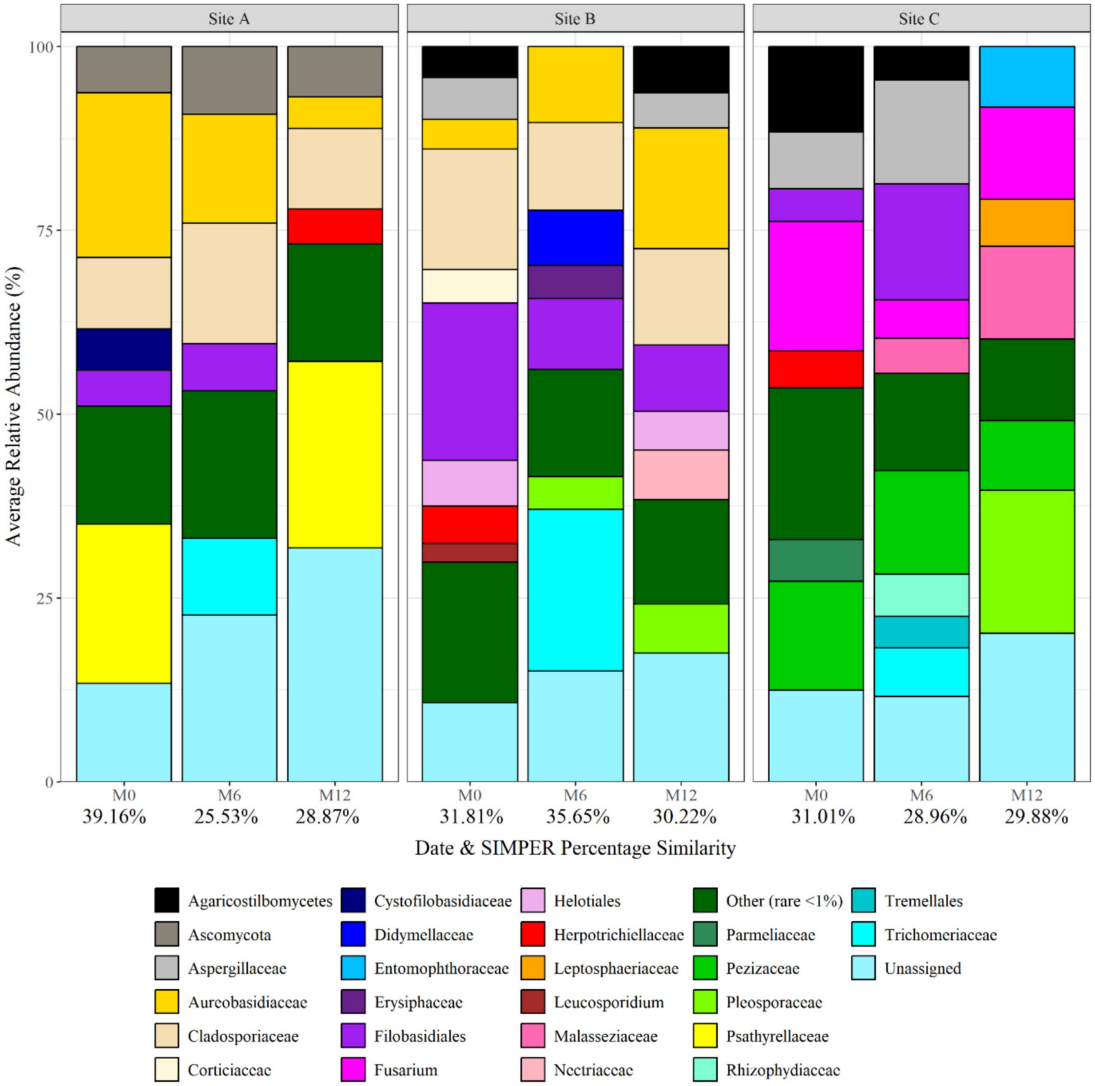
Fungal planktonic community structure at each site and over time at genus level. Numbers below each sampling day (day 0, month 6, and month 12) indicate the average replicate similarity (each bar is the average of three repeat samples). Data responsible for >1% relative abundance plotted.

Figure 5 shows the fungal planktonic community composition at each site, and over time, at genus level. Similar to bacterial relative abundance data, SIMPER analysis of relative abundance data identified that bio-replicates at each sample point were found to be most similar within the water at site C supplied by ground-water (29.88% at month 12), and most different within site A water (28.87% at month 12) (Figure 5). *Pleosporaceae*, *Pezizaceae*, *Psathyrellaceae*, *Cladosporiaceae*, *Aspergillaceae*, *Fusarium*, and *Cladosporiaceae* were abundant across different sites and time points. *Pleosporaceae* was particularly dominant at site C (19.49 average relative abundance at month 12), and *Psathyrellaceae* dominant at site A (25.39 average relative abundance at month 12). SIMPER analysis identified that, of the fungal genera detected, 4, 13, and 15 explained 75% of the difference between the sites at month 0, month 6, and month 12, respectively. Among the differentiating genera were *Aureobasidium*, *Psathyrellaceae*, *Pleosporaceae*, *Herpotrichiellacaea*, *Didymellaceae*, *Trichomeriacaee*, and *Fusarium*, but none were unique to any site or time difference.

### 3.3 Biofilm microbiome

#### 3.3.1 Biofilm bacterial community composition

**Figure 6:**
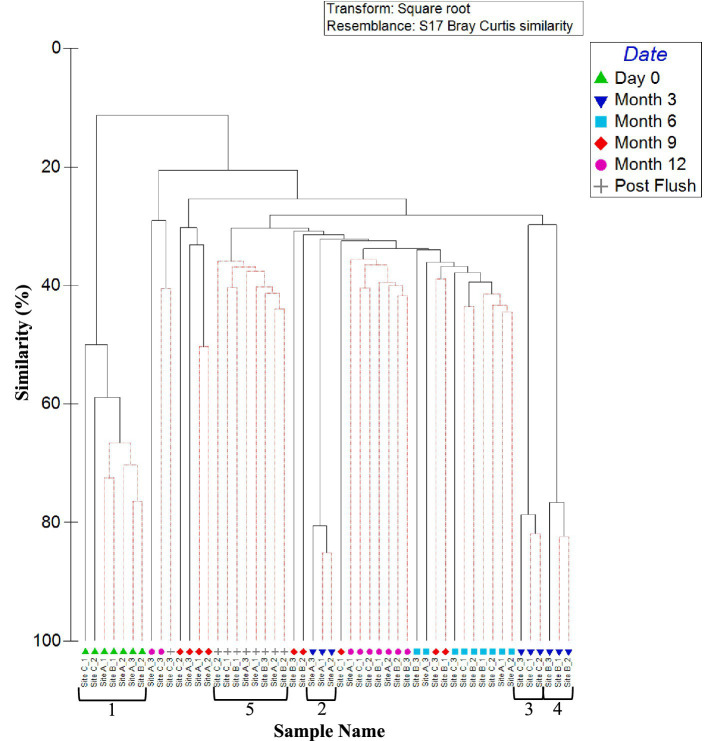
Similarity between biofilm bacterial communities at the three sites over 1 year (including post flush data). Dendrogram based on Bray–Curtis similarities using OTU relative abundance data. Sample names are written in the format of time site_replicate. Time points are day 0, month 3, month 6, month 9, month 12 or post flush as shown in the key. Numbers 1, 2, 3, 4, and 5 highlight distinct clusters. Red lines indicate profiles not significantly dissimilar according to SIMPROF analysis.

Figure 6 shows the similarities in bacterial community between biofilm samples from the three sites and all time points (day 0, month 3, 6, 9, 12, and post-flush), analysed at OTU level. Results do not consistently cluster by site across all timepoints, rather there is evidence of a difference in biofilm bacterial communities at month 3, but then the communities converged. This study found no statistical difference (*p* < 0.05) between microbial communities found on coupons within a pipe loop test facility positioned on the bottom, middle and top of the pipe. This suggests that gravitational processes are not the dominant force acting upon biofilms.

Day 0 samples were found to be independent from all other samples and exhibited no site dependence (day 0 ANOSIM by site: global *R* value = 0.250; *p*-value = 0.143, cluster 1, Figure 6). Month 3 samples were found to cluster independently from all the other samples, with site differences being observed (month 3 ANOSIM by site: global *R* value = 1; *p*-value = 0.004; Clusters 2–4, Figure 6). After 3 months, the impact of site was no longer statistically significant as reflected by a lack of site specific clustering in Figure 6, although there was some clustering of communities by time point. Post-flush samples were found to form another time specific cluster and again no sites specific clustering was observed with no statistically significance (post-flush ANOSIM by site: global *R* value = 0.226; *p*-value = 0.129; Cluster 5, Figure 6).

Month 12 samples from site A and site C showed more variation (30.17% average similarity) between biofilm replicates than site B. This correlates with site B having a chloramine rather than free chlorine disinfection residual, as well as with sites A and C being the high and low (respectively) extremes of other bulk water parameters including cell counts and AOC concentration (Table 2). Twelve months and post flush samples showed some grouping of samples (similarity of 40%), with some clustering by site and time point. Post-flush replicates within a site again showed that biofilm samples from sites A and C were most different (average similarity of 39.21%), therefore the chloramine disinfection residual and less extreme bulk water characteristics (exhibited at site B) were selecting for a more consistent and stable bacterial biofilm community.

**Figure 7:**
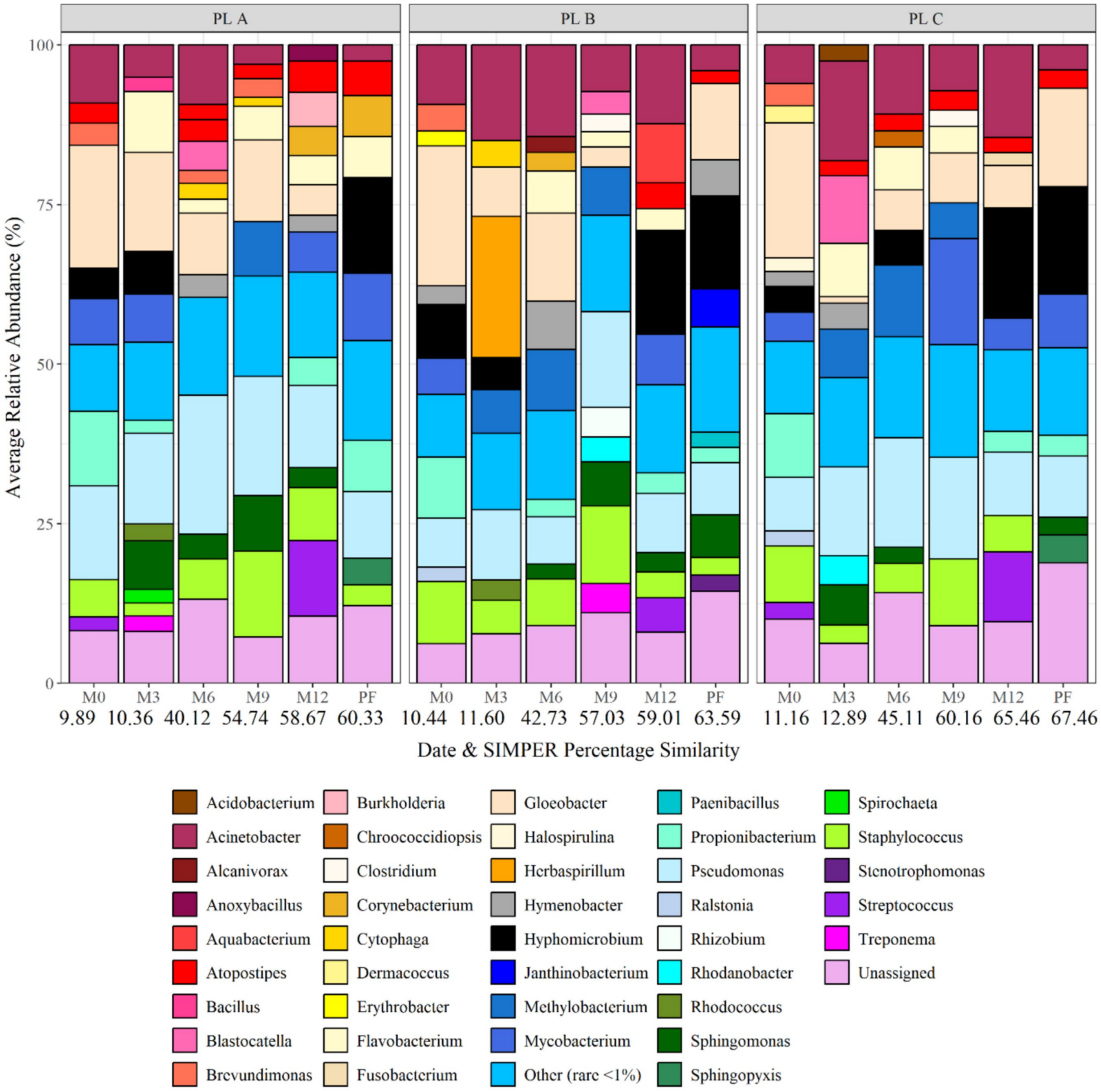
Bacterial biofilm community composition at each site and over time at genus level. Numbers below each sampling day (day 0, month 3, month 6, month 9, month 12, and post flush) indicate the average similarity (calculated using SIMPER analysis) (each bar is the average of three repeat samples). Data responsible for >1% relative abundance plotted.

Figure 7 shows the bacterial biofilm community composition at each site and over time at genus level. The dominant genus at day 0 was *Gloeobacter*, making up 19.31, 21.94 and 21.14% of the average relative abundance at site A, B, and C, respectively. As the biofilms matured, month 3 and month 6 data showed an increase in the relative abundance of *Pseudomonas*, *Staphylococcus*, *Sphingomonas*, and *Acinetobacter*. *Hyphomicrobium* was found to occur within biofilms at month 12 and post-flush, being responsible for 16.86% of the average relative abundance in site C post-flush biofilm samples. SIMPER analysis identified that *Herbaspirillum*, *Atopostipes*, *Hyphomicrobium*, *Corynebacterium*, *Streptococcus*, and *Treponema* were responsible for >75% of the difference in community at month 3. *Herbaspirillum* was abundant in month 3 biofilms at site B (average relative abundance of 22.09%) before being responsible for only <1% of the relative abundance at month 9.

#### 3.3.2 Diversity indices of bacterial and fungal biofilm data

Diversity indices were calculated for bacterial and fungal communities from the biofilm samples taken from pipe loops A, B & C to determine their relative richness, evenness and diversity (Table 5). The relative richness of bacterial communities was significantly different between the three sites at all time points (<0.05) excluding day 0. The relative richness was very low at day 0, before increasing at months 3 and 6 as would be expected for a maturing biofilm. The relative richness of bacterial communities then decreases from 9 months to 12 months, and again decreases further due to flushing, suggesting a loss of specific communities post-flush. This trend in relative richness is similar for all three sites. Bacterial community evenness was found to exhibit no change by site or by time point. Relative diversity followed a similar pattern to relative richness, but was much less pronounced, with an increase to month 9, before dropping at month 12 and again post-flush.

**Table 5:** Ecological indices of biofilm bacterial communities from drinking water biofilm samples taken from pipe loops A, B & C (*n* = 3) at day 0, month 3, month 6, month 9, month 12, and post-flush.

Site	Time	Relative richness (Chao 1)		Relative evenness (Simpson)		Relative diversity (Shannons index)	
		Mean	StDev	Mean	StDev	Mean	StDev
A	Day 0	54.33	8.02	0.93	0.00	3.09	0.07
	Month 3	805.33	44.00	0.98	0.00	4.98	0.10
	Month 6	1017.67	163.37	0.87	0.14	3.76	0.98
	Month 9	1687.67	390.76	0.99	0.01	5.98	0.47
	Month 12	894.33	145.11	0.93	0.03	3.95	0.47
	Post-flush	642.33	167.66	0.96	0.01	4.47	0.10
B	Day 0	52.50	0.71	0.94	0.01	3.15	0.20
	Month 3	1111.00	71.53	0.99	0.00	5.48	0.12
	Month 6	929.33	239.83	0.96	0.02	4.65	0.28
	Month 9	881.33	205.05	0.99	0.00	5.15	0.09
	Month 12	826.00	154.92	0.96	0.01	4.36	0.20
	Post-flush	818.33	55.43	0.94	0.01	4.13	0.05
C	Day 0	57.50	3.54	0.93	0.00	3.16	0.00
	Month 3	1122.67	131.64	0.99	0.00	5.40	0.04
	Month 6	1032.67	118.57	0.98	0.02	4.99	0.33
	Month 9	1043.67	431.62	0.97	0.02	4.91	0.69
	Month 12	940.33	486.46	0.95	0.02	4.12	0.56
	Post-flush	785.67	217.12	0.90	0.03	3.37	0.30

(A) Relative richness, (B) relative evenness, and (C) relative diversity.

The relative richness, relative evenness and relative diversity of fungal communities within drinking water biofilm samples taken from pipe loops A, B & C, are reported in Table 6. Similar to bacterial data, the relative richness and diversity of fungal biofilm communities exhibited an increase from day 0 to month 9 as the biofilms matured with time, before then decreasing at month 12 and post flush.

**Table 6:** Ecological indices of biofilm fungal communities from drinking water biofilm samples taken from pipe loops A, B & C (*n* = 3) at day 0, month 3, month 6, month 9, month 12, and post-flush.

Site	Time	Relative richness (Chao 1)		Relative evenness (Simpson)		Relative diversity (Shannons index)	
		Mean	StDev	Mean	StDev	Mean	StDev
A	Day 0	58.25	0.25	0.90	0.03	2.88	0.23
	Month 3	1024.69	20.02	0.97	0.00	4.43	0.05
	Month 6	1012.69	125.69	0.98	0.01	4.22	0.25
	Month 9	1058.30	127.50	0.97	0.00	4.20	0.11
	Month 12	1004.78	145.33	0.95	0.00	3.49	0.01
	Post-flush	994.60	87.15	0.96	0.01	3.90	0.28
B	Day 0	53.90	0.99	0.90	0.04	2.86	0.30
	Month 3	926.78	88.68	0.97	0.00	4.21	0.09
	Month 6	1157.67	141.21	0.90	0.11	3.72	0.59
	Month 9	1073.04	28.50	0.96	0.01	3.99	0.21
	Month 12	862.13	85.65	0.96	0.01	3.83	0.18
	Post-flush	900.03	39.60	0.85	0.20	3.23	0.98
C	Day 0	62.25	5.30	0.92	0.01	3.11	0.09
	Month 3	1105.10	56.74	0.96	0.02	4.02	0.16
	Month 6	1004.24	128.21	0.97	0.01	4.26	0.41
	Month 9	998.61	172.61	0.93	0.02	3.66	0.22
	Month 12	997.77	185.10	0.90	0.12	3.44	1.39
	Post-flush	851.28	247.54	0.81	0.25	2.81	1.63

(A) Relative richness, (B) relative evenness, and (C) relative diversity.

#### 3.3.3 Biofilm fungal community composition

**Figure 8:**
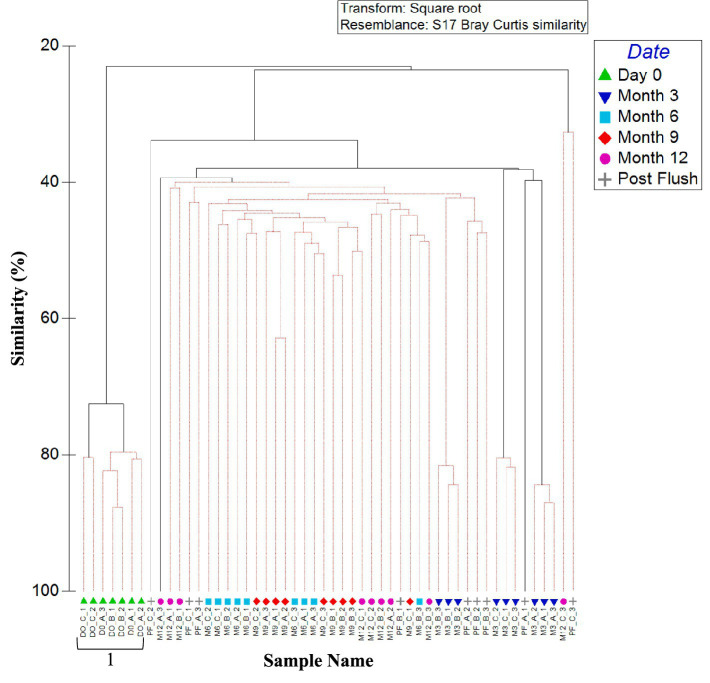
Similarity between biofilm fungal communities at the three sites over 1 year (including post flush data). Dendrogram based on Bray–Curtis similarities using OTU relative abundance data. Sample names are written in the format of time site_replicate. Time points are day 0, month 3, month 6, month 9, month 12 or post flush as shown in the key. Number 1 highlights a distinct cluster. Red lines indicate profiles not significantly dissimilar according to SIMPROF analysis.

The dendrogram in Figure 8 shows the similarities in fungal community between all biofilm samples analysed at the OTU level. Fungi day 0 biofilm samples clustered independently from all other samples (Cluster 1, Figure 8) and exhibited no significant site effect (day 0 ANOSIM by site: global *R* value = 0.55; *p*-value = 0.086). This is similar to what was seen in the bacteria biofilm data, Figure 6. Unlike bacterial biofilm communities, fungi within the biofilm showed no site specific clustering and no site driven distinction between communities at month 3 (ANOSIM global *R* value = 0.25; *p*-value = 0.125). Fungal communities from month 3 onwards did not differ significantly between sample points (Figure 7). This suggests that the fungal community rapidly established and remained relatively stable, and that fungal community composition was independent of site conditions and ongoing (differing) inoculum.

Similarity between replicates of 12 month fungal OTU relative abundance data was again highest at site B (site B month 12 SIMPER: 43.89 similarity). Similarly, 12 month and post flush samples showed some grouping of samples (similarity of 40%). ANOSIM values for month 12 and post-flush fungi OTU relative abundance and presence/absence data again showed high levels of similarity (as indicated by a low global *R* value <0.1, *p* = 0.04) for sites A and B.

**Figure 9:**
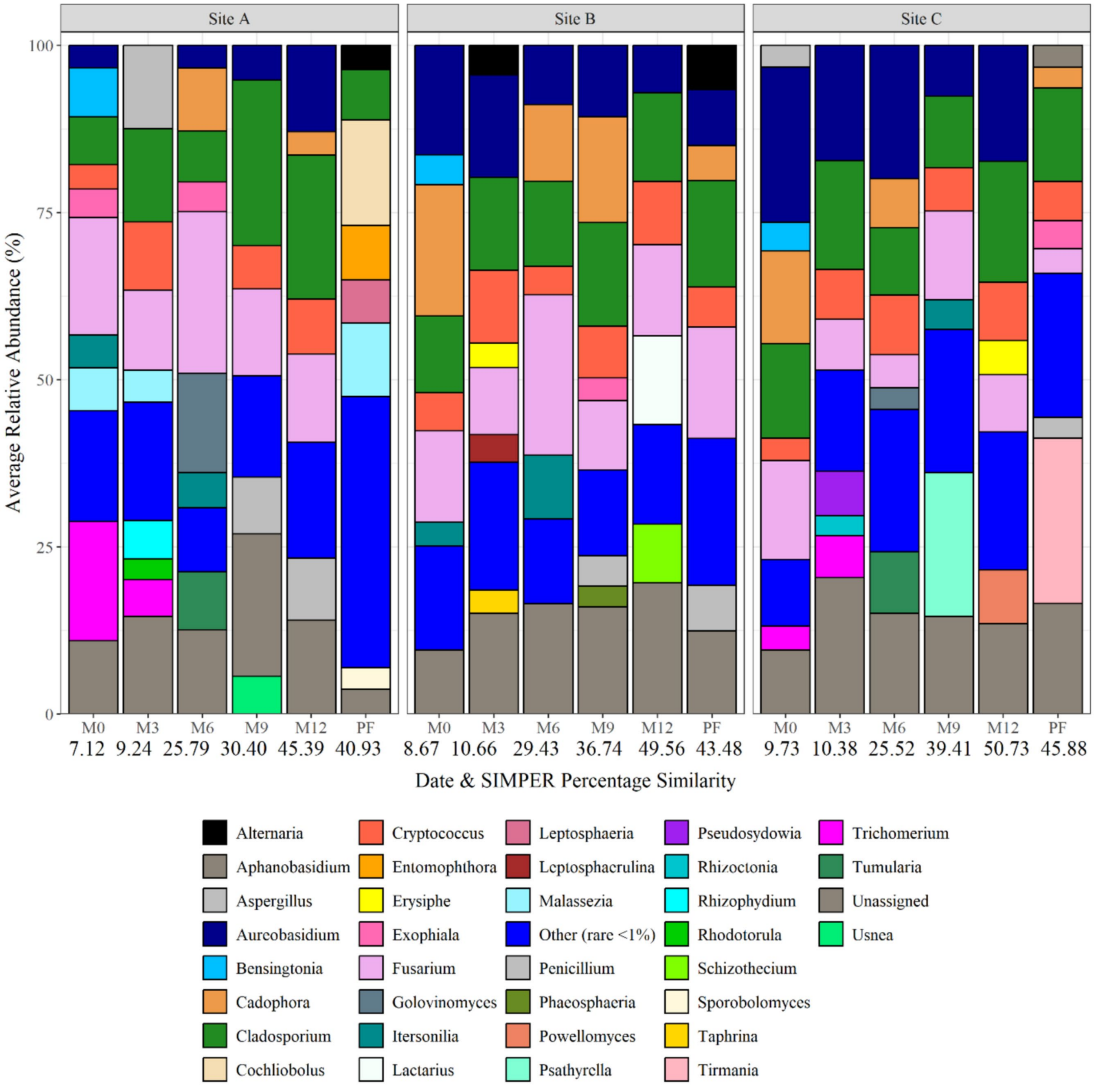
Fungal biofilm community composition at each site and over time at genus level. Numbers below each sampling day (day 0, month 3, month 6, month 9, month 12, and post flush) indicate the average similarity (calculated using SIMPER analysis) (each bar is the average of three repeat samples). Data responsible for >1% relative abundance plotted.

Figure 9 shows the fungal biofilm community composition at each site and over time at genus level. *Fusarium* was the dominant genus at day 0 being responsible for 17.56, 13.67 and 14.79% of the average relative abundance. At month 3, the fungal biofilm communities were dominated by *Cladosporium* at all three sites, followed by *Aspergillus* (12.49%) at site A, *Cryptococcus* (10.92%) at site B and *Aureobasidium* (17.32%) at site C. At month 6, *Cladosporium* remained most abundant within site C (10.06% average relative abundance), with *Fusarium* (24.19) and *Golovinomyces* (14.90%) being dominant in site A and *Fusarium* (24.03) and *Cladosporium* (12.75%) at site B. Biofilm fungal communities became more similar again at month 9 and month 12, with *Cladosporium* being present at all sites. *Psathyrella* became abundant at site C at month 9. Post-flush samples were again domindated by *Cladosporium*, and an increase in *Cochliobolus* (15.78%) and *Malassezia* at site A, *Fusarium* at site B and *Tirmania* at site C.

### 3.4 Comparison of planktonic and biofilm microbiome

**Figure 10:**
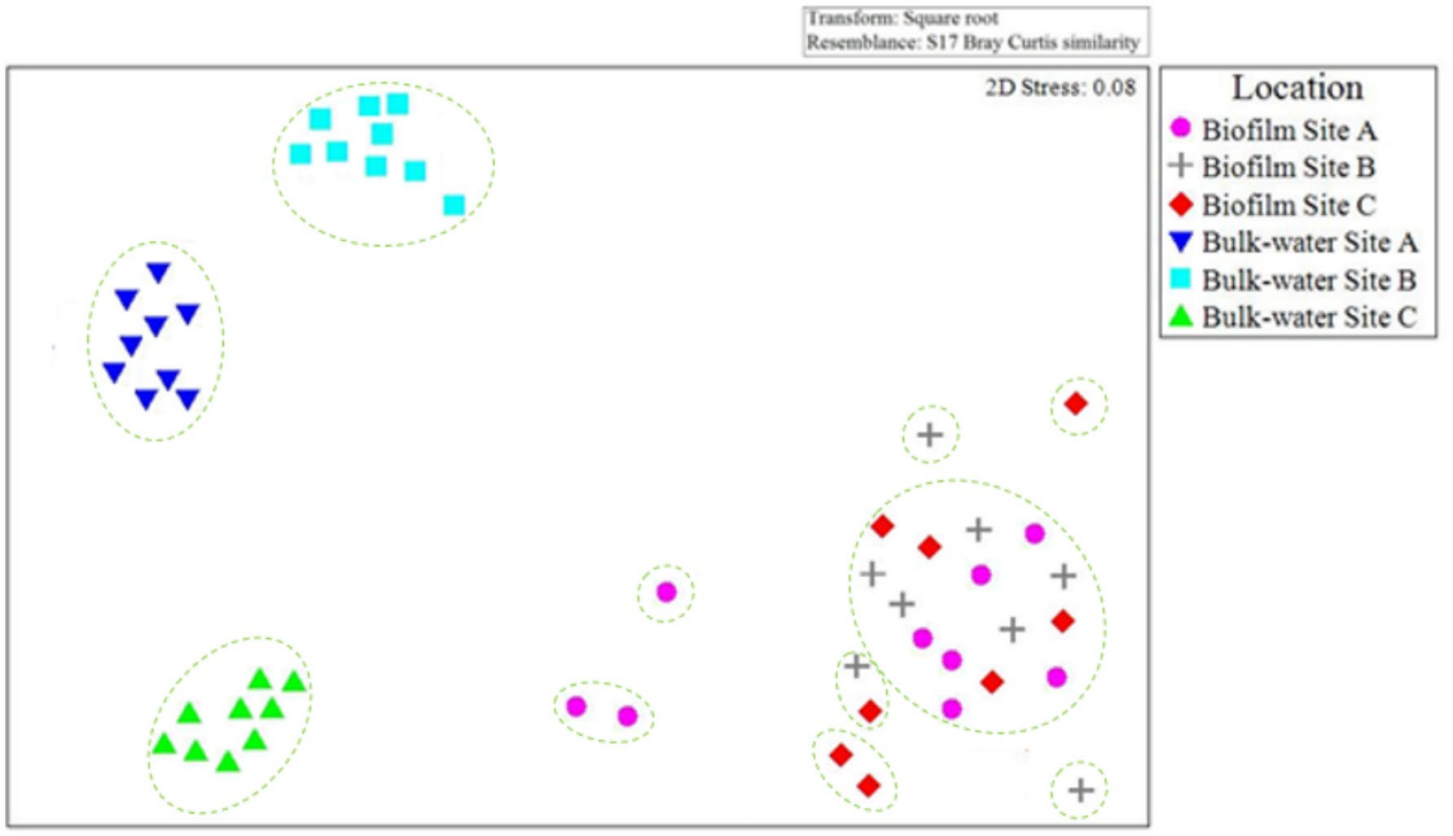
Comparison of planktonic and biofilm bacterial community composition similarities throughout the study at each site A, B, and C. nMDS is based on Bray–Curtis similarities, calculated from OTU relative abundance data for day 0, 6 month, and 12 month bulk-water and biofilm samples. Only 2× day 0 site B and 2× day 0 site C biofilm samples contained quantities of bacterial or fungal DNA detectable via the methods used in this study. Dashed lines indicate clusters of at least 25% similarity based on hierarchical clustering.

The community composition of bacteria and fungi found in the planktonic phase and within the biofilms at sites A, B, and C were compared to evaluate the similarities or differences between samples types within and between sites. Figure 10 presents an nMDS plot comparing planktonic and biofilm bacterial OTU relative abundance data from site A, B, and C for day 0, 6 month, and 12-month time points. The biofilm samples grouped together, independent of site location (Figure 10). In contrast, planktonic water samples clearly clustered by site. ANOSIM 12 month biofilm and planktonic data by site showed that presence/absence data had, on average, a higher global *R* value (*R* = 0.654, *p* = 0.03) than relative abundance data (*R* = 0.325, *p* = 0.04). Similar community bacterial community members were present in the planktonic phase and within the biofilm, but that they were present at different abundances. This suggests that rare or low relative abundance species formed distinct groups, but that these changes were overshadowed by the dominant core species.

**Figure 11:**
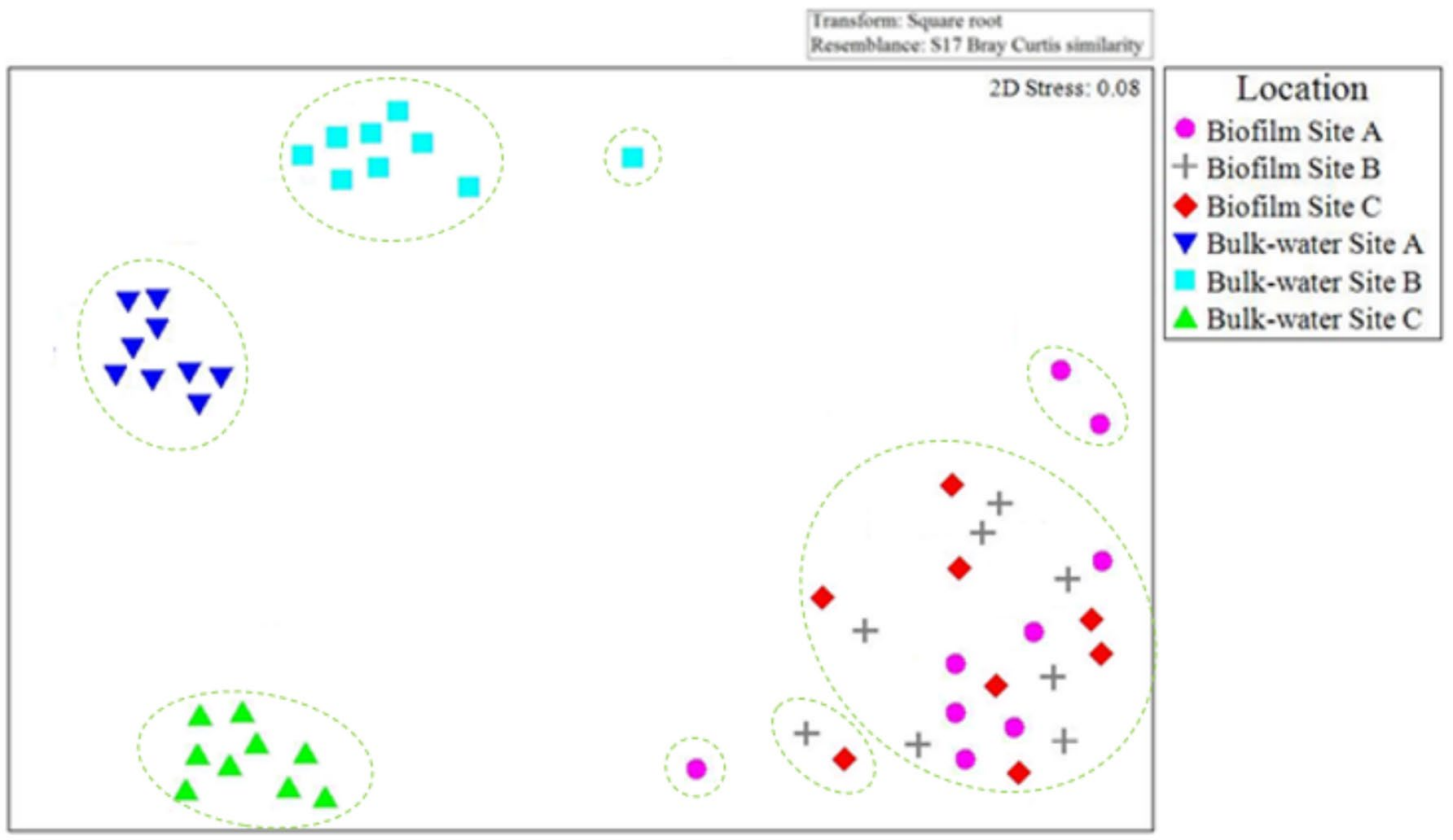
Comparison of planktonic and biofilm fungal community composition similarity throughout the study at each site A, B, and C. nMDS is based on Bray–Curtis similarities of OTU relative abundance data for day 0, 6 month, and 12 month bulk-water and biofilm samples. Only 2× day 0 site B and 2× day 0 site C biofilm samples contained quantities of bacterial or fungal DNA detectable via the methods used in this study. Dashed lines indicate clusters of at least 25% similarity based on hierarchical clustering.

Figure 11 presents an nMDS plot comparing planktonic and biofilm fungi OTU relative abundance data from site A, B, and C day 0, 6 month and 12-month time points. Fungi biofilm samples grouped together, independent of site location (Figure 11) as was seen for the bacteria (Figure 10). Planktonic fungi samples formed distinct clusters as a function of site, similarly to the trend observed with the bacterial communities (Figure 10).

### 3.5 Impact of water quality on community composition

**Figure 12:**
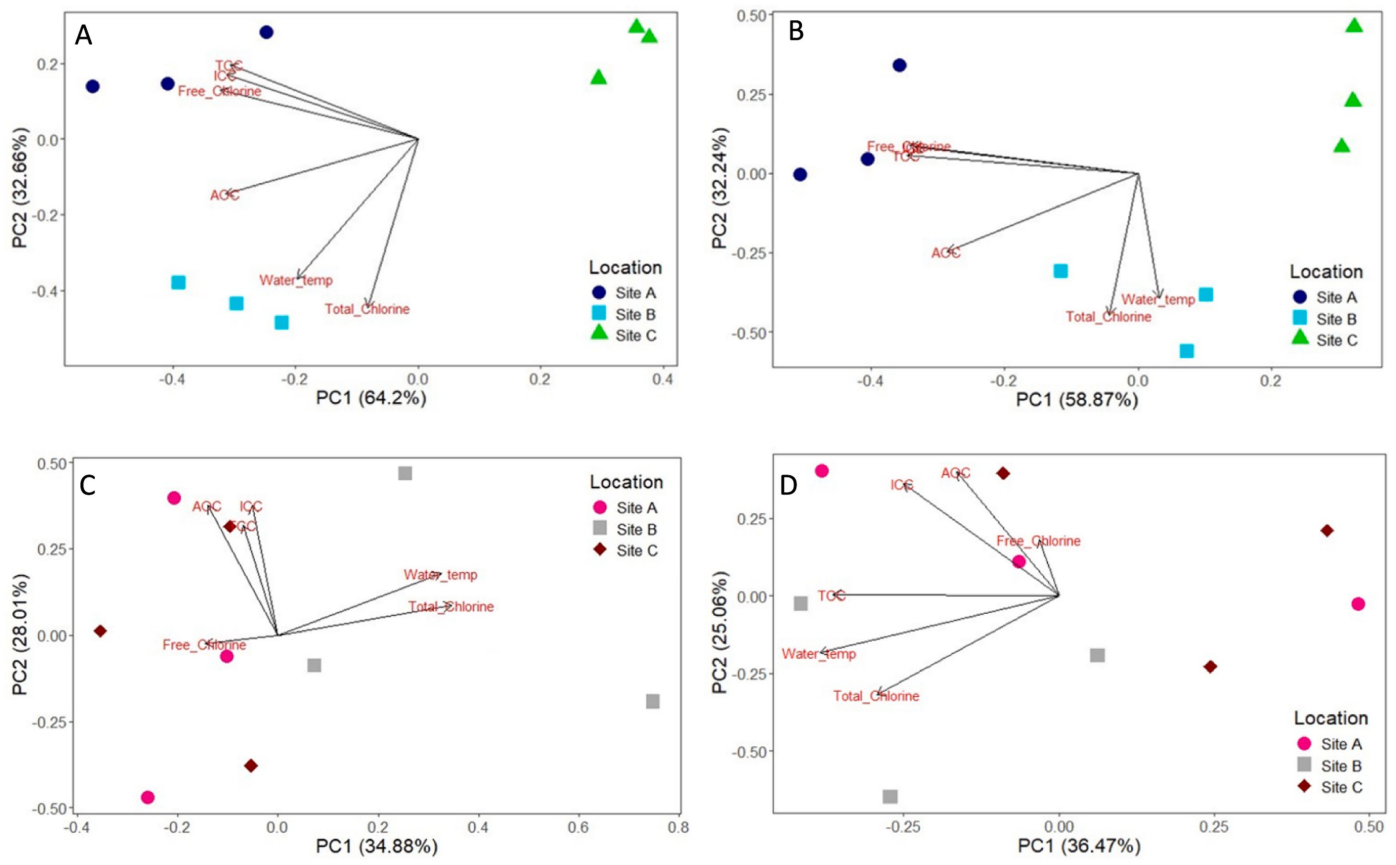
Principal coordinate analysis (PCoA) analysis of the impact of water quality parameters on 12 month bulk water bacteria **(A)** and fungi **(B)**; and 12 month biofilm bacteria **(C)** and fungi **(D)** at site A, B, and C. Samples are coloured according to the site location. Water quality parameters include total chlorine, free chlorine, total cell count (TCC), intact cell count (ICC), assimilable organic carbon (AOC), and water temperature.

The impact of physio-chemical water quality parameters on bulk-water and biofilm community analysis were analysed via principal-coordinate-analysis (Figure 12). PC1 and PC2 explained 32.66–64.2% variation in the bacterial bulk-water and 32.24–58.87% for fungal bulk-water. In contrast, PC1 and PC2 only explained 28.01–34.88% of the variation in biofilm bacteria and 25.08–36.47% of the difference in biofilm fungal communities. Turbidity was not responsible for any differences in bulk-water or biofilm composition. The dominant water quality parameters influencing community composition were chlorine, cell counts, AOC concentration and water temperature.

## 4 Discussion

### 4.1 Planktonic and biofilm community composition comparison

This study investigated the impact of diverse bulk-water quality, including planktonic microbiome assessment, on biofilm community composition within pipe loop facilities over 1 year. The planktonic bacterial and fungal communities found to be at different sites with different water qualities, confirming the observations made by previous studies (Douterelo et al., 2013; Ling et al., 2016). *Acinobacter*, which play a role in degrading organic matter, was found to be unique to the bulk water at sites A and B which are characterised by higher AOC concentrations. In contrast, *Hyphomicrobium* which is suited to oligotrophic environments, was found to be abundant within bulk water at site C, supplied by ground-water.

In contrast, the biofilm community was found to be very similar after three months across the three sites, despite significant differences in the planktonic microbiome, and the incoming water quality (including AOC concentration, disinfection residual, source water and planktonic cell counts). It should be noted that this similarity in biofilm community is in respect to identifying members and not assessing which are active. It is hence likely that the physical conditions of hydraulic regime (Fish et al., 2022), temperature (Li et al., 2018) and pipe material (Henne et al., 2012) were the dominating factors influencing the biofilm microbiome, as these were maintained across the three loops.

### 4.2 Parameters influencing biofilm microbiome similarity

The biofilm communities within these pipe loops were remarkably similar, despite the difference in many bulk-water quality parameters. There were no differences in concentrations of iron, manganese and turbidity between the sites, though these are unlikely to have a dominant impact on community selection (Figure 12). TON and phosphate were also similar between sites (Table 2), and were at concentrations such that neither parameter was likely to be a limiting nutrient (Prest et al., 2016). However, Pick et al. (2021a, 2021b) reported different biofilm growth rates, and different cell count and turbidity responses due to flushing from the same experiment. This was observed to directly correlate with AOC concentration, such that higher AOC concentrations supported increased biofilm cell growth. Hence, while nutrient concentrations are shown here to have little effect on who is present, they did have a stark effect on biofilm quantity with respect to cell concentrations and (in)organic material (particulate metals, primarily iron and manganese, observed as turbidity upon mobilisation from the pipe wall).

#### 4.2.1 Hydraulic conditions

Each of the pipe loops in this study were run at a steady state flow of 0.4 L/s. A constant flow rate was used due to the remote, disparate locations of the sites and to reduce operational risks from feedback control and actuated valves over the one year of operation. In operational DWDS, biofilms are subject to a diurnal flow patterns and exposed to hydraulic variations which influence boundary-layer hydraulics, both shear stress forces experience by the biofilm and turbulence effects for on-going exchange between the pipe wall and bulk-water. Fish et al. (2017) found that biofilm EPS and bacterial communities differed between varied flow (low varied flow and high varied flow) and steady state conditioned biofilms during a 1 month study, despite being supplied by the same water. Furthermore, when analysing the impact of hydraulics on the fungal biofilm community within DWDS, Fish (2013) found that fungi was more common in steady state conditions than low varied flow, and had a distinct community (no fungi was detected in high varied flow after 1 month). Steady state conditions have previously been found to facilitate accumulation of greater biofilm biomass or thicker biofilms than varied flow conditions, as biofilms experienced no hydraulic disturbance during growth (Fish et al., 2017; Fish et al., 2022; Sharpe et al., 2019). This could have potentially impacted the biofilms in this study which were all grown under steady state conditions.

Varying hydraulic conditions have been shown to influence the adhesive/cohesive strength of the biofilm (Khu et al., 2023), the accumulation or mobilisation of biofilms (Fish et al., 2017; Fish et al., 2022; Xin et al., 2024) and the rate of mass-transfer (including nutrients, planktonic cells and disinfectant residual) at the pipe wall. Lehtola et al. (2006) found that the formation of biofilms increased with the water flow velocity within a pilot distribution system, indicating the mass transfer of nutrients (in this case phosphorus was the limiting nutrient) is a major role in the growth of biofilms. However, the relationship between hydraulics and biofilm community composition is complex, with greater flow variation during growth being associated with increased cell quantity but inversely related to EPS-to-cell volume ratios and bacterial diversity in full-scale systems (Fish et al., 2017). This highlights that it is important to consider the influence of hydraulics in combinations with other parameters. It should be noted that the complexities of hydraulic conditions are not fully represented by bench top experimental configurations such as annual reactors, likely explaining why the findings of such experiments often do not translate well to operational systems. In addition to flow rate and flow patterns, the hydraulic residence time of a DWDS will also affect water quality, including nutrient and disinfection residual supply (Shamsaei et al., 2013).

#### 4.2.2 AOC

This study monitored AOC within the bulk-water at each of the three sites, with the AOC concentration being highest at site A and lowest at site C. From the same experiment as reported here, Pick et al. (2021a) found that during flushing AOC release from the biofilm was very similar in each pipe loop independent of bulk-water quality. The explanation offered for this finding was that complex cycling of AOC occurs in the biofilm, in which excess AOC is potentially stored within the biofilm at times of elevated AOC concentration in the bulk-water. Therefore, similar levels of AOC storage within the biofilm could lead to the development of similar community compositions within the biofilm. Nutrients in drinking water follow a gradient towards the pipe wall, with the pipe surface potentially acting a carbon source (Mathews et al., 2023). Proctor et al. (2017) investigated the collective impact of AOC, temperature, and pipe material on microbiota composition and *Legionella pneumophila* in hot water plumbing systems. The influent AOC concentration was found to impact total bacterial numbers but had minimal influence on planktonic opportunistic pathogen gene numbers or microbiota composition. The differences in the bacterial biofilm communities observed in this study up to 3 months, could be due to differences in the AOC concentration in bulk water during biofilm development.

#### 4.2.3 Pipe material

The pipe material used in this study was consistent between each site, so could have been driving similarities in biofilm community. HDPE was used in this study as it is frequently used in modern DWDS (Husband et al., 2008) as it is a high grade plastic with a smooth surface that is stated to have a low biofilm forming potential. However, metals, plastics and cement have all been and still are used in DWDS construction (Fish et al., 2016). The extent to which pipe material impacts community composition is debated, and is also dependent on location within the DWDS and the microorganisms seeding the biofilm (Henne et al., 2012). Henne et al. (2012) found that initial bacterial colonisation within DWDS may be surface-specific but, over time, biofilms become increasingly similar to their neighbouring biofilms.

In contrast, Douterelo et al. (2014) investigated the impact of pipe material (polyethylene and cast iron) on the bacteriological composition of material mobilised from DWDS using distribution management area (DMA) hydrants. The bacterial community composition of the material mobilised from the pipes was significantly different between plastic and cast iron pipe sections, with the highest species richness and diversity being found in the samples from material mobilised from plastic pipes (Douterelo et al., 2014). Further research is required to establish the different pipe materials on drinking water biofilms over longer-term time frames (>3 months), and more representative of biofilms within operational DWDS.

#### 4.2.4 Temperature

The pipe loop experimental facilities used in this study were located within buildings of operational WTW and experienced similar air temperatures. Although there were slightly differences in water temperature between the loops most likely due to seasonal variation in water temperature as a function of the source water (Table 2), biofilms in each pipe loop will have experienced the same rapid heat exchange with the surrounding air. This will have resulting in biofilms in each pipe loop having experienced a relatively constant temperature similar to pipes at an appreciable distance into DWDS (Díaz et al., 2023). Based on this it the biofilms grown in each pipe loop are a good representation of operational systems.

Temperature is known to have an impact on the decay of chlorine facilitating biofilm growth (Li et al., 2018), the incidence of opportunistic pathogens (Ashbolt, 2015), biofilm community composition (Calero Preciado et al., 2021) and biofilm material accumulation and subsequent mobilisation (Fish et al., 2022). Using a pipe loop facility, Calero Preciado et al. (2021) found that large (8°C) temperature variation significantly modified the structure of biofilm microbial communities at the early stages of biofilm development. *Pseudomonas* was found to increase in relative abundance in biofilms developed at 24°C compared to biofilms developed at 16°C, while fungal communities showed loss of diversity and richness, and the increase in dominance of *Fusarium* genus (Calero Preciado et al., 2021). Similarly Fish et al. (2022) reported an impact of temperature variation (8°C vs. 16°C) on biofilm cell quantities, also evidencing the complexity of temperature and hydraulic interactions in governing material accumulation and subsequent mobilisation. This study found that the biofilm bacterial community did exhibit some change over time, whether this being evidence of the community maturing or seasonal changes in temperature. As bacterial biofilm communities were all similar from 6 months growth onwards, this suggests that the communities were not influenced by any spatial or temporal changes in water temperature during that time.

#### 4.2.5 Disinfection residual

It is surprising that the different disinfection residual types and concentrations of the three study sites did not impact the biofilm microbiome beyond month 3. Literature such as Lee et al. (2011) suggest that chloramines penetrate further into biofilms and hence should have a greater impact than free chlorine. Pipe loops A and C were free chlorine systems, while pipe loop B had a chloramine residual (Table 1), and the residual concentrations in each loop were different (Table 2). Month 12 samples biofilm samples from site A and site C showed more variation between biofilm replicates than site B. This suggests that the chloramine residual of site B, selected for a more homogenous bacterial biofilm community. Chloramine has been previously demonstrated to penetrate greater into the biofilm than chlorine and provide better inactivation of cells (Lee et al., 2018). Furthermore, a recent study comparing biofilms from chloraminated DWDS to a DWDS with no residual, found differences between the planktonic and biofilm microbiomes within the systems, as well as differences in biofilm bacterial composition between the disinfected and non-disinfected systems (Waak et al., 2019). Overall the dominant and very surprising result is that the residual types and concentrations did not impact and drive differences in biofilm communities in this study.

#### 4.2.6 Biofilm maturity

A final unique aspect of this research compared to the vast majority of the literature is the duration of the experiments and hence the age of the biofilm, particularly for a fully representative experimental facility. This study was 12 months while many other studies are days to 3 months’ duration. The period for a biofilm to be ‘mature’ is debated with ages cited as mature varying from 385 days (Boe-Hansen et al., 2002), 6–9 months (Pick and Fish, 2024) and 20 years (Henne et al., 2012). Even the definition of a mature biofilm is debated, being cited as quasi-stationary (Boe-Hansen et al., 2002) or dominated by secondary adhesion (Fish et al., 2016) in which the EPS is the main constituent of the biofilm, responsible for binding cells to the infrastructure surface. However, it is likely that the 12 month old biofilms studied here were nearer to a stable condition than others in the literature. In this study, biofilms (bacteria and fungi) were found to cluster independently from all the other samples at 3 months, however after this point there was no site specific clustering but there was some clustering by time point. It is possible that the differences in biofilm community composition observed in other studies (Douterelo et al., 2018; Fish et al., 2020) is evidence of the development or succession of the biofilm community, and that the speed or specific composition of biofilm development is more influenced by conditions such as hydraulics, chlorine residual and nutrient load, rather than the planktonic microbiome.

### 4.3 Practical implications

The lack of influence of the planktonic microbiome on the biofilm microbiome observed here confirms that the planktonic and attached microbiome are distinct. It appears that the inoculum effect from the planktonic community is limited and that the biofilm community is selected as a function of the factors held constant here. It was observed by Pick et al. (2021a) that the nutrient supply, quantified as AOC, has a direct impact of the amount of biofilm, but is observed here not to impact the biofilm microbiome community. AOC input will be defined by and consistent for a given DWDS. Within a given DWDS the pipe material is not readily changed but can be slowly influenced by investment strategies. Hence, of the conserved parameters explored herein, it is the hydraulic regime that remains and can hence be managed to potentially influence who is present in the biofilms of any given pipe in a DWDS.

With prior research, including Fish et al. (2022) showing that hydraulics also impacts the amount of biofilm and its physical structure, as a function of EPS, it is important that we further study the impacts of hydraulics on DWDS biofilm using appropriately representative experimental configuration, be relevant to operational systems and studies for longer than 3 months to enable biofilms to mature. Studies should consider both shear stress, how it impacts on emergent physical cohesive properties, and turbulence, for nutrient supply and ongoing exchange to and from the planktonic community. Within a DWDS it is possible to control the hydraulic conditions of a given pipe through network design, valve operations etc., such that if we knew what a beneficial biofilm was and the hydraulic conditions to promote this we could manage them. Conversely, this research has shown that we do not need to be concerned with the planktonic community of final treated water for DWDS biofilm management.

## 5 Conclusion

This one-year study found that the drinking water biofilm bacterial and fungal communities, were similar after 3 months growth within three pipe loop experimental facilities installed at different operation WTW. This was despite significant differences in planktonic communities, nutrient load and chlorine regime. The pipe loop physical conditions, including pipe material and hydraulic regime, which were consistent between the loops, are hence assumed to be the dominant factor influencing biofilm community composition. This highlights the need to conduct biofilm studies for longer than 3 months in order to accurately assess the impact of different conditions on the drinking water biofilm microbiome.

## Data Availability

The original contributions presented in the study are publicly available. This data can be found here: https://www.ncbi.nlm.nih.gov/, accession number: PRJNA1273313.

## Footnotes

^1^ www.ncbi.nlm.nih.gov
