# Supplementary material for: Similarity of drinking water biofilm microbiome despite diverse planktonic water community and quality
Source: Front Microbiol. 2025 Jun 18;16:1567992. doi: 10.3389/fmicb.2025.1567992 (PMC12213642; doi:10.3389/fmicb.2025.1567992)
Supplement: Supplementary file 1 [file Table_1.docx]

1. **Supplementary Material**

Supplementary Table 1: Bulk-water parameters and analysis methods (after Pick et al 2021).

| **Water Quality Parameter** | **Instrument / Analysis Method** | **Range** | **Resolution** | **Accuracy** |
| --- | --- | --- | --- | --- |
| **AOC^A^** | C6 Flow Cytometer with autosampler (BD Accuri, UK). | 10^2^–10^7^ cells/mL | 10,000 events / second. Sample concentration over 5 x 10^6^ cells / mL | - |
| **TCC^B^ and ICC^C^** | C6 Flow Cytometer with autosampler (BD Accuri, UK). | 10^2^–10^7^ cells/mL | 10,000 events / second. Sample concentration over 5 x 10^6^ cells / mL | - |
| **TOC^D^** | Formacs high temperature catalytic combustion  system (Skalar Analytical B.V., Breda, Netherlands). | 100 ppb^F^ -5000 ppm^G^ carbon | - | - |
| **TON^E^ and Phosphate** | Aquakem 600 Discrete analyser (Thermo Fisher Scientific Ltd., Loughborough, U.K). | Spectral range 340-880 nm. Halogen lamp absorbance range 0-0.25 A | Halogen lamp absorbance resolution of 0.001 A | Halogen lamp absorbance reproducibility of SD ≤0.005A at 2 A. |
| **Iron and Manganese** | PerkinElmer  Nexion 300X ICPMS Spectrometer | - | - | - |
| **Total and Free Chlorine** | Pocket colorimeter (Hach-Lange, Salford, U.K.) | 0.00 to 5.00 mg/L | 0.01 mg/L | ± 0.02 mg/L |
| **Turbidity** | Hach handheld 2100Q formazine calibrated turbidity meter | 0–1000 NTU^H^ | ±1% of reading or 0.01 NTU | ±2% |
| **Online Turbidity** | ATI A15/76 (Analytical Technology Inc, UK) | 0.001 NTU - 4000 NTU | 0.001 NTU | ± 5% of reading or ± 0.02 NTU |
| **Temperature** | Sealey THC100 Thermometer | -50°C to +70°C | - | ±1°C |
| **pH** | ROSS Ultra pH electrode (Thermo Fisher Scientific Ltd.,  Loughborough, U.K.) | 0-14 | - | ± 0.01 |
| **Flow** | Siemens Sitrans fm mag 6000 flow meter | - | - | ± 0.2% of the flow rate |

^A^Assimilable Organic Carbon; ^B^Total Cell Count; ^C^Intact Cell Count; ^D^Total Organic Carbon; ^E^Total Organic Nitrogen; ^F^parts per billion; ^G^parts per million; ^H^Nephelometric Turbidity Units.

Supplementary Table 2: Bulk-water quality within post-treated water during the formation of biofilms over the 12 month period at Site A, Site B and Site C (after Pick et al 2021).

| **Water Quality Parameter** | **Site A**  **Mean (SD^A^)** | **Site B**  **Mean (SD^A^)** | **Site C**  **Mean (SD^A^)** |
| --- | --- | --- | --- |
| TCC (cells/mL)* | 118,668 (133,077) | 1691 (3221) | 450 (1710) |
| ICC (cells/mL)* | 569 (1451) | 206 (2125) | 49 (45) |
| Total Chlorine (mg/l)* | 0.89 (0.11) | 1.34 (0.15) | 0.64 (0.10) |
| Free Chlorine (mg/l)* | 0.80 (0.12) | 0.15 (0.05) | 0.52 (0.06) |
| Turbidity (NTU) | 0.2 (0) | 0.2 (0) | 0.2 (0) |
| Iron (µg/L) | <7^B^ | <7 ^B^ | <7 ^B^ |
| Manganese (µg/L)* | 5.98 (4.09) | 4.96 (3.01) | 2.05 (0.40) |
| AOC (µg C/L)* | 300 (29) | 245 (32) | 73 (13) |
| TOC (mg/L)* | 1.41 (0.31) | 1.49 (0.43) | 0.33 (0.15) |
| Total Organic Nitrogen (mg/L) | 1.22 (0.25) | 1.20 (0.31) | 1.16 (0.30) |
| Phosphate (mg/L) | 0.77 (0.12) | 0.87 (0.26) | 0.84 (0.21) |
| Water temperature (°C)* | 9.12 (5.26) | 9.71 (5.75) | 9.01 (0.79) |

Replication n=3, samples collected fortnightly for 12 months = 72 samples. ^A^SD = standard deviation; ^B^Below the limit of detection. AOC = Assimilable Organic Carbon; TCC = Total Cell Count; ICC = Intact Cell Count; NTU = Nephelometric Turbidity Units; TOC = Total Organic Carbon. Parameters marked with *were statically different between sites.
